# Supplementary material for: The presence and absence of periplasmic rings in bacterial flagellar motors correlates with stator type
Source: eLife. 2019 Jan 16;8:e43487. doi: 10.7554/eLife.43487 (PMC6375700; doi:10.7554/eLife.43487)
Supplement: Supplementary file 1. [file elife-43487-supp1.docx]

**Table S1. Raw T- and H-rings proteins Blast results for all species in Tables 1-4.** For *E*-values exceeding the cutoff, the top hit is listed in parentheses. Note that the term “n.a.” (not applicable) is used if *E*-value does not exceed the cutoff.

| **Species** | **MotX candidate** | **MotY candidate** | **FlgO candidate** | **FlgP candidate** | **FlgT candidate** |
| --- | --- | --- | --- | --- | --- |
| *Azotobacter vinelandii* DJ | n.a. | 8e-14  (Avin_48650) | n.a. | n.a. | n.a. |
| *Cellvibrio japonicas* Ueda107 | n.a. | 9e-28  (CJA_2588) | n.a. | n.a. | n.a. |
| *Chromohalobacter salexigens* DSM 3043 | n.a. | 6e-13  (Csal_3309) | 9e-16  (Csal_2511) | n.a. | n.a. |
| *Pseudomonas entomophila* | n.a. | 2e-31  (PSEEN1209) | n.a. | n.a. | n.a. |
| *Saccharophagus degradans* 2-40 | n.a. | 1e-37  (Sde_2427) | n.a. | n.a. | n.a. |
| *Pseudomonas putida* | n.a. | 7e-31  (PP_1087) | n.a. | n.a. | n.a. |
| *Legionella pneumophila* | n.a. | 3e-35  (lpg2962) | n.a. | n.a. | n.a. |
| *Pseudomonas aeruginosa* | n.a. | 2e-37  (PA3526) | n.a. | n.a. | n.a. |
| *Yersinia pestis CO92* | n.a. | 6e-11  (YPO0448) | n.a. | n.a. | n.a. |
| *Pseudomonas fluorescens Pf0-1* | n.a. | 2e-30  (Pfl01_4518) | n.a. | n.a. | n.a. |
| *Xanthomonas campestris pv. campestris ATCC 33913* | n.a. | 1e-13  (XCC1436) | n.a. | n.a. | n.a. |
| *Xanthomonas axonopodis pv. citrumelo F1* | n.a. | 1e-14  (XACM_1468) | n.a. | n.a. | n.a. |
| *Stenotrophomonas maltophilia R551-3* | n.a. | 4e-10  (Smal_1563) | n.a. | n.a. | n.a. |
| *Escherichia coli* | n.a. | n.a. | n.a. | n.a. | n.a. |
| *Salmonella enterica* | n.a. | n.a. | n.a. | n.a. | n.a. |
| *Sodalis glossinidius* | n.a. | n.a. | n.a. | n.a. | n.a. |
| *Photorhabdus laumondii subsp. laumondii TTO1* | n.a. | n.a. | n.a. | n.a. | n.a. |
| *Serratia proteomaculans* | n.a. | 7e-13  (Spro_1787) | n.a. | n.a. | n.a. |
| *Psychromonas ingrahamii* | n.a. | 3e-14  (Ping_3567) | n.a. | n.a. | n.a. |
| *Colwellia psychrerythraea* 34H | 2e-63  (CPS_4618) | 1e-73  (CPS_3471) | 2e-59  (CPS_1469) | 6e-28  (CPS_1470) | 5e-38  (CPS_1468) |
| *Shewanella oneidensis* MR-1 | 2e-46  (SO_3936) | 2e-80  (SO_2754) | 2e-19  (SO_3257) | 6e-31  (SO_3256) | 3e-36  (SO_3258) |
| *Vibrio fischeri* | 1e-113  (VF_2317) | 3e-141  (VF_0926) | 3e-113  (VF_1884) | 1e-60  (VF_1883) | 2e-166  (VF_1885) |
| *Vibrio vulnificus YJ016* | 4e-136  (VV3065) | 9e-177  (VV1183) | 8e-140  (VV0953) | 1e-77  (VV0954) | 0.0  (VV0952) |
| *Photobacterium profundum* | 1e-110  (PBPRA3344) | 3e-146  (PBPRA2571) | 5e-101  (PBPRA0894) | 5e-60  (PBPRA0895) | 6e-145  (PBPRA0893) |
| *Pseudoalteromonas haloplanktis* | 3e-76  (PSHAa0276) | 6e-73  (PSHAa2115) | 3e-37  (PSHAa0755) | 2e-26  (PSHAa0762) | 5e-40  (PSHAa0761) |
| *Pseudoalteromonas tunicata* | 4e-71  (PTUN_a0699) | 1e-68  (PTUN_a1296) | 2e-32  (PTUN_a3193) | 2e-28  (PTUN_a3178) | 4e-34  (PTUN_a3179) |
| *Idiomarina loihiensis L2TR* | 5e-67  (IL2001) | 4e-78  (IL1801) | 4e-18  (IL1169) | 9e-32  (IL1153) | 1e-30  (IL1154) |
| *Alteromonas macleodii ATCC 27126* | 8e-73  (MASE_16945) | 3e-74  (MASE_05600) | 2e-34  (MASE_11745) | 7e-29  (MASE_04615) | 3e-35  (MASE_04610) |
| *Pseudoalteromonas atlantica* | 2e-71  (Patl_0993) | 1e-79  (Patl_1400) | 1e-30  (Patl_1308) | 1e-26  (Patl_3106) | 1e-31  (Patl_3107) |
