# Supplementary material for: The presence and absence of periplasmic rings in bacterial flagellar motors correlates with stator type
Source: eLife. 2019 Jan 16;8:e43487. doi: 10.7554/eLife.43487 (PMC6375700; doi:10.7554/eLife.43487)
Supplement: Supplementary file 2. [file elife-43487-supp2.docx]

**Table S2. Raw stator proteins Blast results for all species in Tables 1-4.** For *E*-values exceeding the cutoff, the top hit is listed in parentheses. Note that the term “n.a.” (not applicable) is used if *E*-value does not exceed the cutoff.

| **Species** | **MotA** | **MotB** | **MotC** | **MotD** | **PomA** | **PomB** |
| --- | --- | --- | --- | --- | --- | --- |
| *Azotobacter vinelandii* DJ | [avn:Avin_24320](https://www.genome.jp/dbget-bin/www_bget?avn:Avin_24320)  3e-112 | [avn:Avin_24330](https://www.genome.jp/dbget-bin/www_bget?avn:Avin_24330)  2e-92 | [avn:Avin_27650](https://www.genome.jp/dbget-bin/www_bget?avn:Avin_27650)  4e-20 | [avn:Avin_27640](https://www.genome.jp/dbget-bin/www_bget?avn:Avin_27640)  2e-23 | n.a. | n.a. |
| *Cellvibrio japonicas* Ueda107 | [cja:CJA_1170](https://www.genome.jp/dbget-bin/www_bget?cja:CJA_1170)  6e-90 | [cja:CJA_1171](https://www.genome.jp/dbget-bin/www_bget?cja:CJA_1171)  8e-56 | [cja:CJA_2136](https://www.genome.jp/dbget-bin/www_bget?cja:CJA_2136)  6e-114 | [cja:CJA_2135](https://www.genome.jp/dbget-bin/www_bget?cja:CJA_2135)  2e-82 | n.a. | n.a. |
| *Chromohalobacter salexigens* DSM 3043 | [csa:Csal_2026](https://www.genome.jp/dbget-bin/www_bget?csa:Csal_2026)  3e-125 | [csa:Csal_2025](https://www.genome.jp/dbget-bin/www_bget?csa:Csal_2025)  2e-92 | [csa:Csal_1505](https://www.genome.jp/dbget-bin/www_bget?csa:Csal_1505)  1e-26 | [csa:Csal_1504](https://www.genome.jp/dbget-bin/www_bget?csa:Csal_1504)  1e-21 | n.a. | n.a. |
| *Pseudomonas entomophila* | [pen:PSEEN4958](https://www.genome.jp/dbget-bin/www_bget?pen:PSEEN4958)  5e-74 | [pen:PSEEN4957](https://www.genome.jp/dbget-bin/www_bget?pen:PSEEN4957)  1e-59 | [pen:PSEEN3791](https://www.genome.jp/dbget-bin/www_bget?pen:PSEEN3791)  4e-151 | [pen:PSEEN3790](https://www.genome.jp/dbget-bin/www_bget?pen:PSEEN3790)  1e-140 | n.a. | n.a. |
| *Saccharophagus degradans* 2-40 | n.a. | n.a. | [sde:Sde_2159](https://www.genome.jp/dbget-bin/www_bget?sde:Sde_2159)  1e-115  [sde:Sde_3219](https://www.genome.jp/dbget-bin/www_bget?sde:Sde_3219)  3e-40 | [sde:Sde_2158](https://www.genome.jp/dbget-bin/www_bget?sde:Sde_2158)  2e-70  [sde:Sde_3218](https://www.genome.jp/dbget-bin/www_bget?sde:Sde_3218)  4e-24 | n.a. | n.a. |
| *Pseudomonas putida* | [ppu:PP_4905](https://www.genome.jp/dbget-bin/www_bget?ppu:PP_4905)  5e-74 | [ppu:PP_4904](https://www.genome.jp/dbget-bin/www_bget?ppu:PP_4904)  2e-60 | [ppu:PP_4336](https://www.genome.jp/dbget-bin/www_bget?ppu:PP_4336)  7e-149 | [ppu:PP_4335](https://www.genome.jp/dbget-bin/www_bget?ppu:PP_4335)  7e-142 | n.a. | n.a. |
| *Legionella pneumophila* | [lpn:lpg2318](https://www.genome.jp/dbget-bin/www_bget?lpn:lpg2318)  2e-108 | [lpn:lpg2319](https://www.genome.jp/dbget-bin/www_bget?lpn:lpg2319)  7e-67 | [lpn:lpg1781](https://www.genome.jp/dbget-bin/www_bget?lpn:lpg1781)  4e-84 | [lpn:lpg1780](https://www.genome.jp/dbget-bin/www_bget?lpn:lpg1780)  2e-56 | n.a. | n.a. |
| *Pseudomonas aeruginosa* | [pae:PA4954](https://www.genome.jp/dbget-bin/www_bget?pae:PA4954)  2e-77 | [pae:PA4953](https://www.genome.jp/dbget-bin/www_bget?pae:PA4953)  1e-60 | reference | reference | n.a. | n.a. |
| *Yersinia pestis CO92* | [ype:YPO1664](https://www.genome.jp/dbget-bin/www_bget?ype:YPO1664)  0.0  [ype:YPO0746](https://www.genome.jp/dbget-bin/www_bget?ype:YPO0746)  1e-53 | [ype:YPO1665](https://www.genome.jp/dbget-bin/www_bget?ype:YPO1665)  3e-148  [ype:YPO0747](https://www.genome.jp/dbget-bin/www_bget?ype:YPO0747)  5e-40 | n.a. | n.a. | n.a. | n.a. |
| *Pseudomonas fluorescens Pf0-1* | [pfo:Pfl01_0512](https://www.genome.jp/dbget-bin/www_bget?pfo:Pfl01_0512)  1e-71 | [pfo:Pfl01_0513](https://www.genome.jp/dbget-bin/www_bget?pfo:Pfl01_0513)  6e-59 | [pfo:Pfl01_1568](https://www.genome.jp/dbget-bin/www_bget?pfo:Pfl01_1568)  1e-146 | [pfo:Pfl01_1569](https://www.genome.jp/dbget-bin/www_bget?pfo:Pfl01_1569)  2e-140 | n.a. | n.a. |
| *Xanthomonas campestris pv. campestris ATCC 33913* | [xcc:XCC3653](https://www.genome.jp/dbget-bin/www_bget?xcc:XCC3653)  3e-77 | [xcc:XCC3654](https://www.genome.jp/dbget-bin/www_bget?xcc:XCC3654)  3e-59 | [xcc:XCC1891](https://www.genome.jp/dbget-bin/www_bget?xcc:XCC1891)  1e-85 | [xcc:XCC1890](https://www.genome.jp/dbget-bin/www_bget?xcc:XCC1890)  1e-67 | n.a. | n.a. |
| *Xanthomonas axonopodis pv. citrumelo F1* | [xax:XACM_3591](https://www.genome.jp/dbget-bin/www_bget?xax:XACM_3591)  1e-76 | [xax:XACM_3592](https://www.genome.jp/dbget-bin/www_bget?xax:XACM_3592)  1e-56 | [xax:XACM_1940](https://www.genome.jp/dbget-bin/www_bget?xax:XACM_1940)  3e-86 | [xax:XACM_1939](https://www.genome.jp/dbget-bin/www_bget?xax:XACM_1939)  1e-67 | n.a. | n.a. |
| *Stenotrophomonas maltophilia R551-3* | [smt:Smal_0439](https://www.genome.jp/dbget-bin/www_bget?smt:Smal_0439)  2e-85 | [smt:Smal_0438](https://www.genome.jp/dbget-bin/www_bget?smt:Smal_0438)  5e-63 | [smt:Smal_1858](https://www.genome.jp/dbget-bin/www_bget?smt:Smal_1858)  2e-90 | [smt:Smal_1857](https://www.genome.jp/dbget-bin/www_bget?smt:Smal_1857)  5e-69 | n.a. | n.a. |
| *Escherichia coli* | Reference | Reference | n.a. | n.a. | n.a. | n.a. |
| *Salmonella enterica* | [stm:STM1923](https://www.genome.jp/dbget-bin/www_bget?stm:STM1923)  0.0 | [stm:STM1922](https://www.genome.jp/dbget-bin/www_bget?stm:STM1922)  0.0 | n.a. | n.a. | n.a. | n.a. |
| *Sodalis glossinidius* |  |  |  |  |  |  |
| *Photorhabdus laumondii subsp. laumondii TTO1* | [plu:plu1849](https://www.genome.jp/dbget-bin/www_bget?plu:plu1849)  5e-164 | [plu:plu1850](https://www.genome.jp/dbget-bin/www_bget?plu:plu1850)  5e-126 | n.a. | n.a. | n.a. | n.a. |
| *Serratia proteomaculans* | [spe:Spro_2987](https://www.genome.jp/dbget-bin/www_bget?spe:Spro_2987)  8e-168 | [spe:Spro_2986](https://www.genome.jp/dbget-bin/www_bget?spe:Spro_2986)  4e-144 | n.a. | n.a. | n.a. | n.a. |
| *Psychromonas ingrahamii* | [pin:Ping_3580](https://www.genome.jp/dbget-bin/www_bget?pin:Ping_3580)  6e-54 | [pin:Ping_3579](https://www.genome.jp/dbget-bin/www_bget?pin:Ping_3579)  8e-40 | n.a. | n.a. | n.a. | n.a. |
| *Colwellia psychrerythraea* 34H | n.a. | n.a. | [cps:CPS_1524](https://www.genome.jp/dbget-bin/www_bget?cps:CPS_1524)  1e-87 | [cps:CPS_1525](https://www.genome.jp/dbget-bin/www_bget?cps:CPS_1525)  8e-50 | [cps:CPS_1092](https://www.genome.jp/dbget-bin/www_bget?cps:CPS_1092)  2e-124 | [cps:CPS_1093](https://www.genome.jp/dbget-bin/www_bget?cps:CPS_1093)  4e-129 |
| *Shewanella oneidensis* MR-1 | [son:SO_4287](https://www.genome.jp/dbget-bin/www_bget?son:SO_4287)  1e-08 | [son:SO_4286](https://www.genome.jp/dbget-bin/www_bget?son:SO_4286)  9e-16 | n.a. | n.a. | [son:SO_1529](https://www.genome.jp/dbget-bin/www_bget?son:SO_1529)  2e-124 | [son:SO_1530](https://www.genome.jp/dbget-bin/www_bget?son:SO_1530)  5e-125 |
| *Vibrio fischeri* | **n.a.** | **n.a.** | [vfi:VF_A0186](https://www.genome.jp/dbget-bin/www_bget?vfi:VF_A0186)  2e-25 | [vfi:VF_A0187](https://www.genome.jp/dbget-bin/www_bget?vfi:VF_A0187)  5e-26 | [vfi:VF_0714](https://www.genome.jp/dbget-bin/www_bget?vfi:VF_0714)  2e-145 | [vfi:VF_0715](https://www.genome.jp/dbget-bin/www_bget?vfi:VF_0715)  4e-164 |
| *Vibrio vulnificus YJ016* | n.a. | n.a. | n.a. | n.a. | [vvy:VV0871](https://www.genome.jp/dbget-bin/www_bget?vvy:VV0871)  3e-167 | [vvy:VV0872](https://www.genome.jp/dbget-bin/www_bget?vvy:VV0872)  0.0 |
| *Photobacterium profundum* | [ppr:PBPRA0048](https://www.genome.jp/dbget-bin/www_bget?ppr:PBPRA0048)  7e-53 | [ppr:PBPRA0049](https://www.genome.jp/dbget-bin/www_bget?ppr:PBPRA0049)  6e-44 | n.a. | n.a. | [ppr:PBPRA0808](https://www.genome.jp/dbget-bin/www_bget?ppr:PBPRA0808)  5e-143 | [ppr:PBPRA0809](https://www.genome.jp/dbget-bin/www_bget?ppr:PBPRA0809)  2e-171 |
| *Pseudoalteromonas haloplanktis* | n.a. | n.a. | [pha:PSHAa0814](https://www.genome.jp/dbget-bin/www_bget?pha:PSHAa0814)  6e-80 | [pha:PSHAa0815](https://www.genome.jp/dbget-bin/www_bget?pha:PSHAa0815)  3e-42 | [pha:PSHAa2363](https://www.genome.jp/dbget-bin/www_bget?pha:PSHAa2363)  2e-113 | [pha:PSHAa2362](https://www.genome.jp/dbget-bin/www_bget?pha:PSHAa2362)  4e-134 |
| *Pseudoalteromonas tunicata* | n.a. | n.a. | [ptu:PTUN_a3105](https://www.genome.jp/dbget-bin/www_bget?ptu:PTUN_a3105)  2e-82 | [ptu:PTUN_a3104](https://www.genome.jp/dbget-bin/www_bget?ptu:PTUN_a3104)  3e-43 | [ptu:PTUN_a1036](https://www.genome.jp/dbget-bin/www_bget?ptu:PTUN_a1036)  3e-126 | [ptu:PTUN_a1037](https://www.genome.jp/dbget-bin/www_bget?ptu:PTUN_a1037)  6e-122 |
| *Idiomarina loihiensis L2TR* | n.a. | n.a. | n.a. | n.a. | [ilo:IL2135](https://www.genome.jp/dbget-bin/www_bget?ilo:IL2135)  5e-117 | [ilo:IL2134](https://www.genome.jp/dbget-bin/www_bget?ilo:IL2134)  2e-138 |
| *Alteromonas macleodii ATCC 27126* | n.a. | n.a. | n.a. | n.a. | [amac:MASE_12370](https://www.genome.jp/dbget-bin/www_bget?amac:MASE_12370)  5e-125 | [amac:MASE_12365](https://www.genome.jp/dbget-bin/www_bget?amac:MASE_12365)  1e-129 |
| *Pseudoalteromonas atlantica* | n.a. | n.a. | n.a. | n.a. | [pat:Patl_1322](https://www.genome.jp/dbget-bin/www_bget?pat:Patl_1322)  3e-126 | [pat:Patl_1323](https://www.genome.jp/dbget-bin/www_bget?pat:Patl_1323)  6e-128 |
