# Supplementary material for: The presence and absence of periplasmic rings in bacterial flagellar motors correlates with stator type
Source: eLife. 2019 Jan 16;8:e43487. doi: 10.7554/eLife.43487 (PMC6375700; doi:10.7554/eLife.43487)
Supplement: Supplementary file 3. [file elife-43487-supp3.docx]

**Table S3. *S. oneidensis* strains used in this study**

| **Strain** | **Relevant genotype** | **Ref.** |
| --- | --- | --- |
| MR-1 | Wild-type | (Myers and Nealson, 1988) |
| *ΔpilMNOPQ* | type IV pili biogenesis mutant | (Bouhenni et al., 2010) |
| *ΔmshHIJKLMNEGBACDOPQ* | Msh pili biogenesis mutant | (Bouhenni et al., 2010) |
| *ΔpilM-Q, ΔmshH-Q* | mutant that lacks type IV and Msh pili biogenesis genes | (Bouhenni et al., 2010) |
| *Δcrp* | Lacking the cAMP receptor protein (CRP) | (Charania et al., 2009) |
